# Predictors of poor outcome in patients with pulmonary arterial hypertension: A single center study

**DOI:** 10.1371/journal.pone.0193245

**Published:** 2018-04-23

**Authors:** Emilia Stepnowska, Ewa Lewicka, Alicja Dąbrowska-Kugacka, Ludmiła Daniłowicz-Szymanowicz, Paweł Zagożdżon, Rafał Kamiński, Zuzanna Lewicka-Potocka, Paweł Miękus, Dariusz Kozłowski, Wojciech Potocki, Grzegorz Raczak

**Affiliations:** 1 Department of Cardiology, Saint Vincent de Paul Hospital, Gdynia, Poland; 2 Department of Cardiology and Electrotherapy, Medical University of Gdansk, Gdańsk, Poland; 3 Department of Hygiene and Epidemiology, Medical University of Gdańsk, Gdańsk, Poland; 4 Department of Clinical Anatomy, Medical University of Gdańsk, Gdańsk, Poland; 5 1^st^ Department of Cardiology, Medical University of Gdańsk, Gdańsk, Poland; 6 Intercollegiate Faculty of Biotechnology, Medical University of Gdańsk, Gdańsk, Poland; Scuola Superiore Sant'Anna, ITALY

## Abstract

**Objectives:**

Pulmonary arterial hypertension (PAH) is a rare disorder with unfavorable prognosis despite implementation of specific PAH-oriented therapy. The aim of the study was to define predictors of poor prognosis in patients from one center treated according to the Polish National Health Fund program.

**Patients and methods:**

Forty-seven consecutive patients (30 women; aged 39±17 years) with PAH diagnosis were enrolled to the study. Clinical assessment, laboratory measurements, electrocardiogram, echocardiography, 6-minute walk test, 24-hour Holter monitoring, cardiopulmonary exercise test and microvolt T-wave alternans test were performed during routine visits. Eight patients died during 2.6±1.7 years follow-up.

**Results:**

Parametrs which differentiated patients who died were brain natriuretic peptide (BNP) concentration ≥330 pg/mL (sensitivity 88%, specificity 92%, area under the ROC curve [AUC] 0.92); bilirubin concentration ≥1.2 mg/dL (sensitivity 88%, specificity 81%, AUC 0.85); right atrial area ≥21 cm2 (sensitivity 86%, specificity 69%, AUC 0.84), right ventricular (RV) dimension in the apical 4-chamber view ≥47 mm (sensitivity 86%, specificity 86%, AUC 0.85) and RV to left ventricular diastolic diameter ratio ≥1.5 (sensitivity 83%, specificity 84%; AUC 0.85). In multivariate analysis, independent predictors of mortality were higher BNP (p = 0.04) and bilirubin level (p = 0.03), higher right atrial area (p = 0.02) and lower tricuspid annular plane systolic excursion (p = 0.03).

**Conclusions:**

In PAH patients treated with specific PAH-oriented therapy right atrial enlargement, impaired right ventricular systolic function, as well as increased BNP and bilirubin concentration was associated with an increased mortality risk.

## Introduction

Pulmonary arterial hypertension (PAH) is a severe medical condition of varying etiology. Prognosis in PAH remains serious even after implementation of specific therapy and drugs that target different pathologic pathways of the disease. In Poland, specific drug therapy is carried out in accordance with the recommendations of the National Health Fund program.

In the recent ESC guidelines a multidimensional approach has been proposed in patients with PAH and a comprehensive prognostic evaluation was discussed in detail [1]. The present study aimed to investigate prognostic factors of poor outcome in an unselected population of patients with PAH from a single Polish center who were all receiving PAH-specific drug therapy at the time of the study. Moreover, it investigated whether microvolt-level T-wave alternans (MTWA) testing provides new information in terms of risk assessment in PAH patients. MTWA testing was reported as a non-invasive method to assess the risk of ventricular arrhythmias in patients with cardiomyopathies of various etiology [2–6]. In PAH mainly the right ventricle (RV) must be concerned with T-wave formation and thus possible MTWA phenomenon. However, until now only a few studies were conducted on MTWA in patients with PAH, in whom hypertrophy and/or dilatation of RV, and not the left ventricle, predominates.

## Methods

### Study group

This was a prospective observational study that included all adult patients with PAH treated according to the Polish Health Fund program in the Department of Cardiology Medical University of Gdansk. This program was introduced in Poland in 2009 and included patients with a definite diagnosis of PAH and RV failure of at least WHO class III. The first line treatment according to the Polish Health Fund program consisted of bosentan or sildenafil. The second line therapy was introduced in case of heart failure progression to WHO class IV or signs of poor prognosis in control RHC (right atrial pressure >10 mmHg, cardiac index <2.4 l/min/m^2^ or mixed venous blood saturation ≤65%). It included bosentan, inhaled iloprost or subcutaneous treprostinil together with sildenafil. Epoprostenol was not available in Poland during the study period. Patients with vasoreactive PAH were treated with calcium channel blockers. Before 2009 only a few of our patients were treated with sildenafil at their own expense.

The diagnosis of PAH had been established by right heart catheterization (RHC) on the presence of an increase in mean pulmonary arterial pressure ≥25 mmHg at rest and pulmonary artery wedge pressure ≤15 mmHg in the absence of other causes of precapillary pulmonary hypertension [1]. Thus patients with pulmonary hypertension due to left heart disease, lung diseases, thromboembolic or other rare disorders were excluded. The date of PAH diagnosis corresponded to the date of confirmatory RHC. Patients with Eisenmenger syndrome and a confirmatory RHC in the childhood did not need to have the RHC repeated before implementation of the PAH specific treatment.

The study protocol was approved by the Independent Ethics Committee of Medical University in Gdansk (NKBBN/447/2015-2016) and all patients gave their written informed consent for participation in the study. Previously diagnosed patients with PAH who were at least 3 months under PAH-specific drug therapy were consecutively enrolled from June 2011 to January 2016. All planned tests were performed during scheduled follow-up visits. Patients treated with calcium channel blockers were not incorporated in the study. Routine evaluation included PAH medical history and co-morbidities, assessment of the WHO functional class, physical examination and non-encouraged 6-minute walk test (6MWT), performed according to the American Thoracic Society recommendation [7]. Renal function impairment was recognized if the estimated glomerular filtration rate (eGFR) was <60 mL/min, calculated using the Cockcroft-Gault formula [8]. In each patient, electrocardiography (ECG), 24-hour Holter monitoring, echocardiography, cardiopulmonary exercise test (CPET) and, if eligible, MTWA testing was performed, and blood samples were taken.

### Laboratory measurements

Blood samples were taken for complete blood count, creatinine, electrolytes, liver enzymes, total bilirubin, iron, uric acid, and plasma brain natriuretic peptide (BNP) level. BNP level was measured using the Abbott Architect assay (Abbott Diagnostics).

### Transthoracic echocardiography

Cardiac chamber dimensions and the right and left ventricular function were evaluated using a commercially available system (Vivid E9, GE Healthcare, Horten, Norway) and a 3.5-MHz phased transducer. All images were stored for further off-line analysis using EchoPac workstation (version BT12, GE Healthcare, Norway). Left ventricular (LV) diameters were measured at end-systole and end-diastole from the parasternal long-axis and 4-chamber views. The LV end-systolic and end-diastolic volumes were calculated from the apical 2- and 4-chamber views, and LV ejection fraction (LVEF) was subsequently calculated by the Simpson’s biplane method. Depth-adjusted two-dimensional (2D) LV images were acquired from the apical 2-, 3- and 4-chamber views for off-line measurements of biplane LV global longitudinal strain (LV GLS) using 2D speckle-tracking echocardiography. Frame rates were optimized at the time of acquisition to 45 frames/s. To assure accuracy and reproducibility, systole was defined as the interval between aortic valve opening and closure, as measured using pulsed-wave Doppler sampling of blood flow in the LV outflow tract. Endocardial border at the LV end-systole was manually selected in the appropriate imaging plane. It was verified whether peak systolic strain from each LV segment was measured prior to aortic valve closure. The LV GLS was calculated as the average of peak strain values of 17 LV segments in the apical 2-, 3- and 4-chamber views. The presence of LV diastolic dysfunction was also recognized, which was defined as at least impaired LV relaxation (grade I LV diastolic dysfunction). In order to establish the presence of the LV diastolic dysfunction the left atrial (LA) volume index (normal value <34 mL/m^2^), the E to A ratio from the mitral inflow profile in baseline conditions and after Valsalva maneuver and average E/e’ ratio were assessed [9]. Normal diastolic function was recognized when LA was not enlarged, the E to A ratio was ≥0.8 and E/e’ ratio was ≤8. Grade I dysfunction was present if E to A ratio was ≤0.8 and E/e’ ratio was ≤8. Grade II dysfunction was diagnosed if LA was enlarged, the E to A ratio was 0.8 to 1.5 with its decrease of ≥50% or an increase in A-wave velocity during the Valsalva maneuver (not caused by E and A fusion) and E/e’ ratio 9 to 14. Grade III dysfunction was present if LA was enlarged, E to A was ≥2 and E/e’ >14.

Right ventricular (RV) function was analyzed in detail according to the EACVI guidelines [10]. Echocardiographic measurements included the right atrial area (in the apical 4-chamber view at end-systole) and RV size (end-diastolic basal diameter in the apical 4-chamber view) Right ventricular systolic pressure was calculated on the basis of peak tricuspid regurgitant jet velocity and an estimate of right atrial (RA) pressure, which was done on the basis of inferior vena cava (IVC) size and its respiratory collapse. In the setting of IVC diameter <2.1 cm that collapsed >50% with a sniff right atrial pressure was estimated as normal (5 mmHg), whereas if IVC was diameter >2.1 cm and collapsed <50% with a sniff high RA pressure was assumed (15 mmHg). In scenarios in which IVC diameter and collapse did not fit this paradigm, an intermediate value of 10 mmHg was used [11].

The RV systolic function was evaluated based on tricuspid annular plane systolic excursion (TAPSE), RV fractional area change (RVFAC), tricuspid lateral annular systolic velocity (RV S’), acceleration of isovolumic contraction (IVC Tacc) assessed by spectral tissue Doppler imaging (TDI), and biplane RV global longitudinal strain (RV strain) measured using 2D speckle-tracking echocardiography. The RV strain was calculated as the average of peak strain values of three segments of the RV free wall and three segments of the interventricular septum. The presence of pericardial effusion was also noted.

### Cardiopulmonary exercise test (CPET)

A symptom-limited CPET was performed under continuous 12-lead ECG monitoring (CardioDirect 12) using a cyclo-ergometer (Lode Corival, Lode B.V., Netherlands). Heart rate and blood pressure were measured automatically (SunTech Tango, Sun Tech Medical, USA) at rest, during each stage of exercise, and at peak exercise. Starting with 20W and increasing the load by 10W every minute, oxygen uptake (VO_2_), carbon dioxide output (VCO_2_), expiratory gas concentrations throughout the respiratory cycle, and minute ventilation (VE) were measured continuously on a breath-by-breath basis using the Cortex equipment with Metasoft 3.9 software (Cortex Biophisik Gmbh, Germany). The peak oxygen consumption (VO2peak) was derived from the maximum average value during 30 seconds at maximal endurance. The anaerobic threshold was calculated using the V-slope method and corrected using the ventilator equivalent method. Pulmonary gas exchange was assessed based on VE/VCO2 slope and percutaneous oxygen saturation.

### MTWA testing

MTWA testing was performed in patients in sinus rhythm (subjects with atrial fibrillation or ventricular pacing were not subjected to MTWA testing). All prescribed medications, including beta-blockers, were continued prior to the test. Three orthogonal Frank leads X, Y, Z (High-Res electrodes, Cambridge Heart-Spacelabs Healthcare, Snoqualmie, WA, USA), as well as 12 standard precordial leads were placed. Treadmill exercise test (Delmar Reynolds, Cambridge Heart-Spacelabs Healthcare, Snoqualmie, WA, USA) was performed using a previously described protocol for MTWA testing [12]. MTWA was analyzed using the analytic spectral method (Cambridge Heart-Spacelabs Healthcare, Snoqualmie, WA, USA). Standard criteria were used in the interpretation of the tests, and the result was categorized as positive, negative, or indeterminate [13]. Indeterminate results were categorized as resulting from patient-related factors (inability to reach the target HR of 105–110 bpm, frequent ventricular premature beats exceeding 10% of the recording, non-sustained alternans) or technical reasons (artifacts resulting from a high level of noise). Indeterminate tests due to noise were repeated immediately, and if determinate, the result of the repeated test was used in the analysis.

### Statistical analysis

Data are presented as mean ± standard deviation (SD), median and range, or a number and percentage of patients. The analyzed parameters were compared between two groups of patients: those who died and those who survived the follow-up period. The Student’s *t* test for independent samples was used for normally distributed continuous variables, and the Mann-Whitney U test was used if variables did not follow a normal distribution. Differences between categorical variables were evaluated using the chi-square test. We performed a receiver operating characteristic curve analysis to investigate the clinical value of selected parameters with respect to the occurrence of death. To identify characteristics associated with a poor outcome, the logistic regression analysis was performed. Variables with P≤0.2 by univariate analysis were included in the multivariate analysis but in case of co-linearity between two or more variables only one of them was included in the multivariate model. For this reason, LVEF showing co-linearity with LV GLS was not included in the multivariate analysis, and only one parameter (TAPSE) describing RV systolic function was taken into account. P<0.05 was considered statistically significant. All statistical analyses were performed using the STATA software (version 12.1, STATACorp).

## Results

The study group included 47 patients with PAH, including 30 women and 17 men, at the mean age of 39±17 years. The time from the first RHC that revealed PAH to the beginning of the study was (median) 4 years. The causes of PAH included congenital heart disease and Eisenmenger syndrome in 26 patients, connective tissue disease in 4 patients (systemic lupus erythematosus, systemic sclerosis, dermatomyositis, and mixed connective tissue disease each in one patients), and idiopathic PAH in 17 patients. At the time of study initiation, all patients received specific drug therapy for PAH, including sildenafil in 19 patients, bosentan in 27 patients, iloprost in 5 patients, and treprostinil in 3 patients. Combined therapy was used in 11 patients. The time of PAH-specific therapy before the initiation of the study was (median) 7.2 months (range 4 months to 3.9 years).

The mean WHO functional class in the study population was 2.7±0.6 (range 1–4), and the 6-minute walk test distance was 386±119 m. In laboratory measurements the mean BNP concentration was 255±558 pg/mL, and total bilirubin 1.1±0.6 mg/dL. During spiroergometric testing, the mean duration of exercise was 5±2.2 min, VO_2_ peak was 10.7±2.4 mL/kg/min, and VO_2_ AT was 8.1±2.2 mL/kg/min.

Echocardiography showed RV dilatation (44±11 mm in the apical 4-chamber view), significantly increased RV systolic pressure (86±25 mmHg), and borderline parameters of RV systolic function: TAPSE 18±6mm, RV S’ 11.1±3 cm/s, RVFAC 34±10%, and RV GLS -18.8±-8%. Right atrial area was 20±8 cm^2^. Notably, a high RV to LV end-diastolic diameter ratio of 1.3±0.4 was found. Pericardial effusion was noted in 15 patients. Regarding LV systolic function, LVEF was normal at 60±9%, while LV GLS was slightly reduced at -17.6±-4%.

Holter monitoring showed >100 premature ventricular beats/24 hours in 19 patients, and nonsustained ventricular tachycardia in 3 patients. MTWA testing result was positive in 27 patients, negative in 14 patients, and indeterminate in 6 patients.

**Fig 1:**
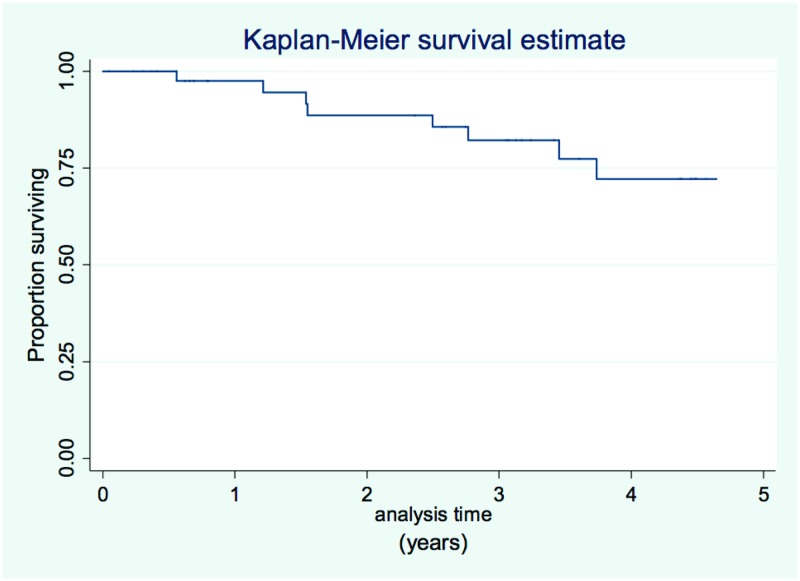
Survival since the time of study initiation (n = 47).

**Fig 2:**
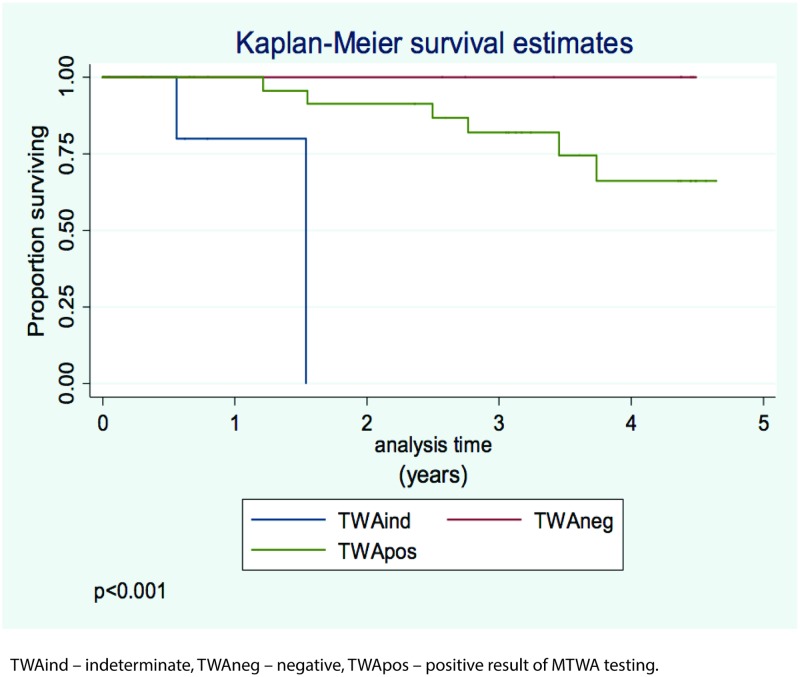
Survival in the study population in relation to the microvolt T-wave alternans (MTWA) testing result. TWA ind- microvolt T-wave alternans indeterminate, TWAneg-microvolt T-wave alternans negative, TWApos- microvolt T-wave alternans positive.

The mean duration of the follow-up was 2.6±1.7 years. During this time, 8 patients died, including 4 due to progression of heart failure, 2 patients due to sudden cardiac death, and the remaining 2 patients due to infective complications (pneumonia). Fig 1 shows survival in the study population, and Fig 2 shows patient survival in relation to MTWA testing result.

The study population was divided into two groups based on the occurrence of death for any reason. Overall, 8 patients died and 39 patients survived the follow-up period.

The two groups did not differ in regard to age, gender distribution, etiology of PAH, severity of heart failure symptoms, concomitant conditions, and the treatment used, including specific drug therapy for PAH (Table 1). There was no difference in time of PAH-specific therapy before the study initiation between the groups. We also did not find differences in spiroergometric testing and Holter monitoring (Table 2). However, the patients who died during the follow-up had lower systolic blood pressure, and higher BNP, total bilirubin, and alkaline phosphatase levels at baseline (Table 1). Among those who died, a positive MTWA testing result was found in 6 patients, and an indeterminate result in 2 patients (Table 2). Among those who survived, a positive MTWA testing result was found in 21 patients, an indeterminate result in 4 patients, and a negative result in 14 patients (P = 0.06).

**Table 1:** Baseline clinical characteristics of patients with pulmonary arterial hypertension (PAH) who died or survived during follow-up.

Variable	Patients who died (n = 8)	Patients who survived (n = 39)	P
Age (years)	43±19	38±16	0.4
Female/male (n/%)	5(63)/3(37)	25(64)/14(36)	1.0
**Diagnosis (n/%)**:			
Idiopathic PAH	4(50)	13(33)	0.4
Connective tissue disease	1(12)	3(8)	0.5
Congenital heart disease	3(38)	23(59)	0.4
History of syncope (n/%)	3(38)	11(28)	0.7
WHO functional class	3.0±0.7(2–4)	2.6±0.6(2–3.5)	0.3
**Co-morbidities (n/%)**:			
Systemic hypertension	2(25)	4(10)	0.3
Diabetes mellitus	0	3(7)	1.0
Hypothyroidism	0	8(20)	0.3
Renal impairment	2(25)	5(12)	0.6
Paroxysmal/persistent AF	2(25)	2(5)	1.0
**Physiological measurements**:			
HR (beats/min)	84±15	86±15	0.7
SBP (mmHg)	101±18	115±12	**0.03**
DBP (mmHg)	68±19	76±13	0.2
Pulse pressure (mmHg)	34±15	39±13	0.4
**Laboratory measurements**:			
BNP (pg/mL)	898±1138	116±131	**<0.001**
Hemoglobin (g/dL)	16.2±3.6	17.3±3.7	0.5
PLT (x 10^3^/uL)	188±95	165±58	0.4
Sodium (mmol/L)	138±2	140±3	0.2
Iron (μg/dL)	75±48	80±46	0.8
Uric acid (mg/dL)	7.5±3	7.3±2	0.9
Total bilirubin (mg/dL)	1.7±0.7	1.0±0.4	**0.002**
GGT (U/l)	67±43	48±40	0.06
ALP (U/l)	96±21	74±24	**0.02**
AST (U/l)	30±8	26±10	0.1
ALT (U/l)	19±6	25±14	0.4
Creatinine (mg/dL)	1±0.3	0.8±0.1	0.1
6MWT (m)	332±172	398±104	0.1
**Cardiopulmonary exercise test**:			
VO_2_peak (mL/kg/min)	10.5±1.4	10.8±2.5	0.8
VO_2_AT (mL/kg/min)	8±1.9	8.1±2.3	0.9
VE/VCO_2_ slope	49±14	42±13	0.3
SBP max (mmHg)	121±26	135±20	0.06
Decrease in SaO_2_ (n/%)	5(62)	18(46)	0.1
Exercise duration (min)	6±3.6	4.8±1.9	0.3
**PAH-specific treatment at the time of the study** (n/%):			
Bosentan	4(50)	23(59)	0.7
Sildenafil	5(62)	14(35)	0.2
Inhaled iloprost	1(12)	4(10)	1.0
Subcutaneous treprostinil	1(12)	2(5)	0.4
Combined therapy	3(38)	8(20)	0.4
PAH-specific treatment before enrollment (years)	1.35±1.36	0.97±1.05	0.9
[median]	0.58	0.52	
**Conventional therapy at the time of the study** (n/%)			
Diuretics	6(72)	19(48)	0.2
Digoxin	2(25)	1(2)	0.07
Beta-blocker	2(25)	7(17)	0.6
ACEI/ARB	2(25)	3(7)	0.2
Warfarin	3(37)	13(33)	1.0
Time from the first RHC that revealed PAH (years)	3.6±3.1	11.2±11.4	0.3
[median]	[3.1]	[5.5]	
Follow-up (years)	2.2±1.1	2.7±1.7	0.4
[median]	[2.0]	[3.1]	

WHO-World Health Organization, AF–atrial fibrillation, HR–heart rate, SBP–systolic blood pressure, DBP–diastolic blood pressure, VO_2_peak–peak O_2_ uptake, VO_2_AT–O_2_ uptake measured at anaerobic threshold, VE/VCO2 –ventilatory equivalent for VCO_2_, SaO_2_–oxygen saturation, SBP max–peak systolic arterial pressure, BNP–brain natriuretic peptide, PLT–platelet count, GGT–gamma-glutamyltranspeptidase, ALP–alkaline phosphatase, ALT–alanine aminotransferase, AST–aspartate aminotransferase, 6MWT–6-minute walk test, ACEI–angiotensin-converting enzyme inhibitor, ARB–angiotensin receptor blocker, RHC–right heart catheterization.

**Table 2:** Baseline electrocardiographic characteristics of patients in the two study groups.

Variable	Patients who died (n = 8)	Patients who survived (n = 39)	P
**ECG**			
QRS duration (ms)	104±14	103±11	0.7
RV hypertrophy (n/%)	5(62)	21(53)	0.4
**24-hour Holter monitoring**:			
Minimal HR (beats/min)	54±12	50±11	0.4
Maximal HR (beats/min)	123±19	127±16	0.5
Mean HR (beats/min)	76±10	77±10	0.9
Patients with >100 premature atrial beats (n/%)	2(25)	9(23)	0.4
Patients with >100 premature ventricular beats (n/%)	4(50)	15(38)	0.06
Patients with nsVT (n/%)	2(25)	1(2)	1.0
**T-wave alternans result (n/%)**:			
Positive	6(75)	21(53)	0.06*
Negative	0	14(35)	
Indefinite	2(25)	4(10)	

ECG–electrocardiogram; HR–heart rate, RV–right ventricle, nsVT–non-sustained ventricular tachycardia.

*Fisher’s test.

As both groups were characterized by a wide range of time since last RHC, the results of RHC were not included in our group comparison. Among those who died, median time since last RHC was 1.6 years (range 1 month to 7.3 years) compared to 1.5 years (12 days to 33 years) among those who survived.

Echocardiography showed significantly higher RV dimension, higher RV to LV diastolic diameter ratio, and higher right atrial area in those patients who died. This group was also characterized by significantly worse RV systolic function parameters including TAPSE, RV S’, RVFAC, and RV strain, and significantly worse LV systolic function as evaluated based on LVEF and LV GLS (Table 3).

**Table 3:** Baseline echocardiographic characteristic of patients in the two study groups.

Variable	Patients who died (n = 8)	Patients who survived (n = 39)	P
RVEDD (mm)	52±5	42±11	**0.02**
RVWT (mm)	10±2	9.6±2	0.5
RV: LV	1.7±0.4	1.2±0.4	**0.004**
Right atrial area (cm^2^)	29±11	19±6	**<0.001**
Tricuspid regurgitant velocity (cm/s)	4.3±0.8	4.3±0.7	0.9
RVSP (mmHg)	86±25	86±26	0.9
TAPSE (mm)	13±5	18±5	**0.01**
RV S’ (cm/s)	8.2±3	11.5±3	**0.02**
RVFAC (%)	26±11	36±10	**0.03**
RV strain (%)	-18±-5	-24±-18	0.07
IVC Tacc (m/s^2^)	2±0.5	2.7±1.2	0.1
LVEF (%)	52±10	62±8	**0.002**
LVESD (mm)	24±9	27±7	0.5
LVEDD (mm)	37±7	43±7	0.3
LVESV (mL)	24±14	27±19	0.5
LVEDV (mL)	52±18	70±36	0.2
IVS (mm)	11±2	11±2	0.4
PWD (mm)	11±2	10±2	0.4
Interventricular delay (ms), median	12	12	0.4
LV GLS (%)	-12±-2	-18±-4	**0.001**
LV diastolic dysfunction (number of pts/%)	6(75)	22(56)	0.4
Pericardial effusion (number of pts/%)	1(12)	14(36)	0.4

RVEDD–right ventricular (RV) end-diastolic diameter in the apical 4-chamber view, RVWT–RV wall thickness, RV:LV–RVEDD to LVEDD ratio (measured in the 4-chamber apical view), RVSP–RV systolic pressure, TAPSE–tricuspid annular plane systolic excursion, RV S’–tissue Doppler-derived tricuspid lateral annular systolic velocity, RVFAC–RV fractional area change, LVEF–left ventricular ejection fraction, LVESD–left ventricular end-systolic diameter, LVEDD–left ventricular end-diastolic diameter, IVS–interventricular septum thickness, PWD–posterior wall thickness, IVC Tacc–tissue Doppler-derived time of acceleration of isovolumic contraction, LV GLS–left ventricular global longitudinal strain in speckle tracking strain analysis.

**Fig 3:**
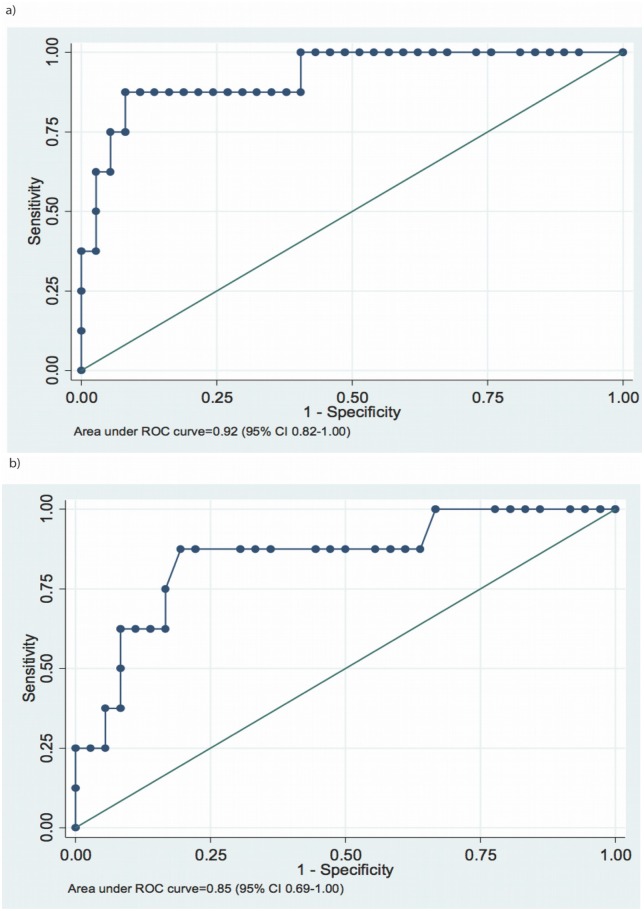
Receiver-operating characteristic curves for a) brain natriuretic (BNP) and b) serum total bilirubin concentration, for separation of patients who died from those who survived during the follow-up period.

In the overall study population of patients with PAH, we analyzed which of the evaluated parameters could differentiate those who died from those who survived. One of these parameters was BNP level ≥330 pg/mL (sensitivity 88%, specificity 92%, area under the ROC curve [AUC] 0.92, 95% confidence interval [CI] 0.82–1.00) (Fig 3a). Also bilirubin level ≥1.2 mg/dL differentiated between those who died and those who survived (sensitivity 88%, specificity 81%, AUC 0.85, 95% CI 0.69–1.00) (Fig 3b).

**Fig 4:**
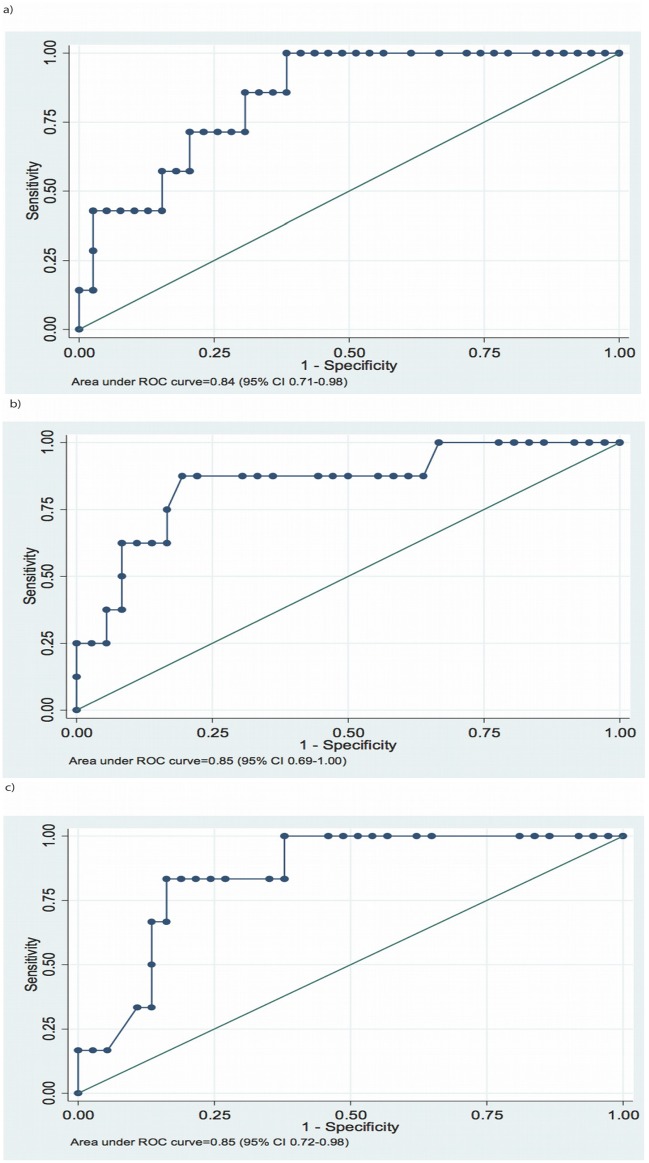
Receiver-operating characteristic curves for a) right atrial area, b) right ventricular end-diastolic diameter, measured in the apical 4-chamber view, and c) the ratio of the right ventricular to the left ventricular end-diastolic diameter (in the 4-chamber apical view) by echocardiography, for separation of patients who died from those who survived during the follow-upperiod.

We also found that those who died could be differentiated from those who survived based on the right atrial area ≥21 cm^2^ (sensitivity 86%, specificity 69%, AUC 0.84, 95% CI 0.71–0.98) (Fig 4a), the RV dimension in the apical 4-chamber view ≥47 mm (sensitivity 86%, specificity 86%, AUC 0.85, 95% CI 0.73–0.97) (Fig 4b), and the RV to LV diastolic diameter ratio ≥1.5 (sensitivity 83%, specificity 84%; AUC 0.85, 95% CI 0.72–0.98) (Fig 4c).

In univariate analysis, independent predictors of mortality included higher BNP and bilirubin levels, higher right atrial area and RV dimension, and lower TAPSE, RV S’, RVFAC, and RV strain. In multivariate analysis, independent predictors of mortality included higher BNP and bilirubin levels, higher right atrial area, and lower TAPSE (Table 4).

**Table 4:** Predictors of mortality in the study population.

Variables	Univariate	P	Multivariate	P
	OR (95% CI)		OR (95% CI)	
BNP (pg/mL)	1.001 (1.000–1.002)	0.002	1.002 (1.000–1.004)	0.04
Bilirubin (mg/dL)	4.14 (1.65–10.40)	0.002	17.38 (1.32–229.60)	0.03
Right atrial area (cm^2^)	1.08 (1.02–1.14)	0.007	1.24 (1.04–1.48)	0.02
RVEDD (mm)	1.79 (1.11–2.89)	0.02	0.50 (0.05–5.43)	0.6
TAPSE (mm)	0.77 (0.63–0.93)	0.007	0.54 (0.31–0.95)	0.03
RV S’ (cm/s)	0.59 (0.41–0.86)	0.006		
RVFAC (%)	0.87 (0.78–0.97)	0.01		
RV strain (%)	0.91 (0.81–1.01)	0.04		

Abbreviations see Tables 1 and 3

## Discussion

Assessment of risk in patients with PAH has a major effect on therapeutic management and thus on the disease course. In the present study, we evaluated this issue in a group of patients with PAH who all received specific drug therapy in accordance with the therapeutic program, which is underway in Poland. In this study, we showed that RV function determined survival in the study population. Worse RV function, as evidenced by lower TAPSE, higher BNP level, and enlarged right atrium, was associated with an increased mortality risk. Higher total bilirubin level, which might be related to worse metabolic function of the liver due to impaired RV systolic function, was also associated with worse outcomes. We also found that patients with a negative MTWA testing result seem to have better outcome, as no one with a negative result died during the follow-up period.

The 2015 ESC guidelines [1] on the diagnosis and management of pulmonary hypertension [1] indicated a number of factors that allow estimation of annual mortality risk in patients with PAH. The presence of pericardial effusion and right atrial enlargement are significant prognostic factors based on echocardiography. In our study population, pericardial effusion was identified in 15 patients but it was not shown to affect outcome. In contrast right atrial area ≥21 cm^2^ differentiated between those who died and those who survived. In the ESC guidelines, the right atrial area associated with intermediate and high annual mortality risk was estimated at 18–26 cm^2^ and above 26 cm^2^, respectively. In the study by Raymond et al. [14], the cut-off value for a higher mortality risk was 20 cm^2^. In the study by Austin et al. [15], increased mortality risk was associated with the right atrial area above 18 cm^2^.

In our study, we found a significantly higher RV dimension and worse RV systolic function, as evidenced by lower TAPSE, RVFAC, RV S’ and RV strain, in those patients who died. These observations are in agreement with findings reported by other authors. The RV dimension of ≥47 mm, and the RV to LV diastolic diameter ratio of ≥1.5 in the apical 4-chamber view differentiated between patients who died and those who survived. Other studies also underlined the prognostic importance of RV dilation in patients with PAH [16] but few of them included the RV to LV diameter ratio (which is normally below 0.66). Tricuspid annular systolic excursion, which can be measured relatively easily in comparison to the other mentioned RV systolic function parameters, was shown to correlate with RV ejection fraction [17, 18]. In our study, lower TAPSE was an independent predictor of mortality during the mean follow-up of 2.6 years. Steiner et al. [19] showed that TAPSE below 18 mm was associated with an increased mortality risk in patients with PAH, while Kjaergard et al. [20] showed a similar association for TAPSE below 14 mm. Ghio et al. [21] found higher survival in patients with TAPSE above 15 mm with absent or only mild tricuspid regurgitation. Of note, the prognostic values of TAPSE is reduced with concomitant severe tricuspid regurgitation [22] or LV dysfunction [23].

Of other RV parameters, reduced RVFAC was found to be associated with worse outcomes in patients with PAH [21,24]. Austin et al. [15] found that RVFAC below 35% was associated with worse patient survival. Other authors highlighted the importance of impaired RV longitudinal strain as evaluated using 2D speckle-tracking. Sachdev et al. [25] found that RV strain ≥-12.5% was associated with an increased mortality risk. Haeck et al. [26] showed that patients with RV strain ≥-19% had more severe symptoms (higher WHO functional class) and lower TAPSE. In addition, RV strain ≥-19% was associated with an increased mortality risk during 2.6 years of follow-up (HR 3.40; P = 0.02). In our study, however, RV strain value between the group who died and those who survived did nor reach statistical significance. Speckle-tracking echocardiography seems an important imaging technique in patients with PAH. Vitarelli et al. [27] showed that evaluation of global and segmental RV function using three-dimensional echocardiography and 2D and 3D speckle-tracking better reflects RV haemodynamics and systolic function compared to conventional echocardiography.

In our study, another independent predictor of mortality in patients with PAH was increased BNP level. The serum level of this polypeptide reflects mostly the fraction released by ventricular cardiomyocytes in response to pressure overload, and to a lower extent the fraction released by atrial cardiomyocytes. In our study population, BNP level ≥330 pg/mL differentiated between patients who died and those who survived the follow-up period. The prognostic importance of natriuretic peptides in patients with PAH and their utility for monitoring treatment effects was indicated in multiple studies [28–30]. In the study by Nagaya et al. [31] in a group of 60 patients with idiopathic PAH, baseline BNP level ≥180 pg/mL and its further increase during only 3 months of follow-up was associated with unfavorable outcomes, despite the use of prostacyclines in 82% of patients. Park et al. [32] also showed that regardless of baseline BNP level, its increase at 3 months of epoprostenol therapy was associated with a risk of heart failure worsening or death, while a reduction in BNP level by more than 50% indicated a favorable disease course. In the 2015 ESC guidelines [1], it has been highlighted that annual mortality risk in PAH patients with BNP level above 300 ng/L exceeds 10%.

Chronically increased RV and right atrial pressure, resulting in impaired hepatic venous outflow, may impair metabolic function of the liver. This malfunction may be reflected by increased bilirubin level. In our study, we showed that augmented bilirubin level was an independent predictor of poor outcomes, and level ≥1.2 mg/dL differentiated between patients who died and those who survived. These observations are in agreement with findings of other authors. In a group of 37 patients with PAH, Takeda et al. [30] showed that total bilirubin level ≥1.2 mg/dL was an independent predictor of mortality risk (HR 13.31, P<0.001). Patients with elevated bilirubin level had more severe symptoms of heart failure (higher WHO class), higher BNP level, worse RV systolic function by echocardiography, and higher right atrial pressure during cardiac catheterization. In 237 patients with idiopathic PAH, Xu et al. [33] evaluated direct bilirubin level at baseline and after specific PAH treatment continued for the mean of 8.3 months. Similarly to our study, these authors found that baseline bilirubin level was an independent predictor of mortality during 40 months of follow-up. Additionally, in those who survived bilirubin level decreased under PAH-specific treatment, which was not seen in patients who died.

In our study, we extended previous research on MTWA in patients with PAH. In one of the first studies an abnormal MTWA testing result was found in 64% patients with PAH [34]. Until now, MTWA was mostly tested in patients with LV systolic dysfunction or LV heart failure to predict the risk of sudden death. The most recent 2014 EHRA/HRS/APHRS guidelines on the prevention of sudden cardiac death, however, did not confirm the utility of MTWA testing for that purpose [35]. The importance of MTWA in patients with PAH is unclear, particularly due to the fact that high mortality in this group is mostly due to heart failure resulting from progressive RV dysfunction, while sudden deaths due to malignant ventricular arrhythmias are relatively rare. Nevertheless, few studies evaluated arrhythmia and the mechanisms of sudden death in patients with PAH [36,37]. Similarly to previous studies, we found that a negative MTWA testing result is rare in patients with PAH—in our study a negative MTWA result was found in 30% of patients, but none of them died. Holter monitoring showed frequent ventricular extrasystole (more than 100 beats per 24 hours) in 19 patients, and unsustained ventricular tachycardia in 3 patients, but no significant differences were noted between the patients who died and those who survived during the follow-up period. We also did not find differences in QRS duration and the rate of ECG criteria of RV hypertrophy, with only a trend for a positive or indeterminate MTWA testing result (P = 0.06). However, we found interesting that patients with a negative MTWA result may have better outcome, as there were no deaths in patients with a negative result. Nevertheless, this observation requires confirmation in a larger PAH population. Moreover, it would be interesting to study changes in MTWA results as a part of the natural history of PAH, as well as under the PAH-specific therapy. This may allow to determine definitively the prognostic significance of MTWA testing in PAH patients.

Our results confirmed previous observations [38] that those patients who died were characterized not only by a worse RV systolic function but also by a worse LV systolic function, as evidenced by a significantly lower LVEF, but first and foremost by lower LV GLS. The importance of this observation in the context of Holter monitoring and MTWA testing results is unclear. Both, ventricular arrhythmia and an abnormal MTWA testing results are usually associated with LV dysfunction. In PAH, however, it is the RV that becomes hypertrophied and dilated. Abnormal MTWA testing result in PAH patients is a frequent and interesting observation but determining whether it indicated LV systolic dysfunction or the risk of ventricular arrhythmia in this patient population requires further studies.

As mentioned above, we consider it interesting to continue the study on prognostic significance of MTWA testing in PAH population and its usefulness when deciding to escalate treatment. Moreover, in further studies on prognosis in PAH, it is important to refer to the individual parameters tested whether the patient is already being treated with PAH-specific therapy or has just started treatment. For example, elevated BNP level, RV enlargement and dysfunction will have different significance in a patient who is just starting a specialized treatment and in a patient who is already under a combined therapy. In addition, important prognostic information in PAH patients has been recently obtained through magnetic resonance imaging (MRI). With increased availability of this technique, this may contribute to better risk stratification in this patient population, including appropriate early treatment escalation.

## Limitations of the study

The present study included a small study sample and was heterogeneous in regard to the etiology of PAH. However, it was a single-center study that included consecutive patients. We did not evaluate changes in the analyzed parameters during the follow-up. Among the analyzed variables, right heart catheterization parameters were not included. In many patients this diagnostic study was performed many years earlier and at various disease stages, which limited the utility of right heart catheterization parameters for predicting the risk. Moreover, intravenous prostanoids were not available in Poland at the time of the study, which might have influenced the results, as this therapy has shown mortality benefits in PAH patients.

## Conclusions

In patients with PAH receiving specific drug therapy, RV function is the main determinant of survival. Right atrial enlargement, reduced TAPSE, higher BNP and bilirubin levels predicted increased mortality in this patient population. Patients with a negative MTWA testing result seem to have better outcome but this observation requires further study.

## Supporting information

S1 File (XLSX)
